# Pan-cancer analysis of frequent DNA co-methylation patterns reveals consistent epigenetic landscape changes in multiple cancers

**DOI:** 10.1186/s12864-016-3259-0

**Published:** 2017-01-25

**Authors:** Jie Zhang, Kun Huang

**Affiliations:** Department of Biomedical Informatics, The Ohio State University, Columbus, OH 43210 USA

**Keywords:** DNA co-methylation, Pan-cancer methylation, Frequent network mining, Epigenetics

## Abstract

**Background:**

DNA methylation is the major form of epigenetic modifications through which the cell regulates the gene expression and silencing. There have been extensive studies on the roles of DNA methylation in cancers, and several cancer drugs were developed targeting this process. However, DNA co-methylation cluster has not been examined in depth, and co-methylation in multiple cancer types has never been studied previously.

**Results:**

In this study, we applied newly developed lmQCM algorithm to mine co-methylation clusters using methylome data from 11 cancer types in TCGA database, and found frequent co-methylated gene clusters exist in these cancer types. Among the four identified frequent clusters, two of them separate the tumor sample from normal sample in 10 out of 11 cancer types, which indicates that consistent epigenetic landscape changes exist in multiple cancer types.

**Conclusion:**

This discovery provides new insight on the epigenetic regulation in cancers and leads to potential new direction for epigenetic biomarker and cancer drug discovery. We also found that genes commonly believed to be silenced via hypermethylation in cancers may still display highly variable methylation levels among cancer cells, and should be considered while using them as epigenetic biomarkers.

**Electronic supplementary material:**

The online version of this article (doi:10.1186/s12864-016-3259-0) contains supplementary material, which is available to authorized users.

## Background

DNA methylation is the most extensively studied form of epigenetic modification in the cell. The reversible addition of a methyl group to the cytosine residue on large clusters of CpG dinucleotides (called CpG islands) can result in chromatin structural changes or physical barriers for proteins binding to DNA [1]. Therefore DNA methylation level regulates gene transcription activities and expression levels, exerting an important role in programming cell development and differentiation [1]. The genome-wide DNA methylation pattern (DNA methylome) is affected by cell age, tissue types, and many environmental factors such as nutrients and carcinogen exposure [2]. Aberrant DNA methylation pattern is a hallmark of cancer [3], and it has been speculated that DNA methylation change may play a role in cancer initiation, development [4], and drug resistance [5, 6]. Studies on DNA methylome in cancers have been a focus of cancer research for more than a decade [7]. It is generally believed that in cancer cells tumor suppressor genes are hypermethylated in the promoter region and are repressed, while oncogenes are hypomethylated and abnormally active [8]. Researchers applied this notion to cancer drug designs, and developed cancer drugs targeting DNA methyltransferases, attempting to correct the abnormal methylation pattern of tumor suppressors and oncogenes [9]. Also quite a few genes have been associated with certain phenotypes (CpG island methylator phenotype, CIMP [7, 10, 11]) or identified as prognosis/diagnosis biomarkers in cancer due to the ease of detection and analysis in body fluid, and the events may happen in the early or premalignant stage of tumor development [1].

Currently, the studies on multiple DNA methylome in cancers are mostly carried out as differential methylation analysis or supervised/unsupervised clustering to look for distinct methylation signature on a specific type of cancer [12, 13]. There is a lack of systematic study on genome-wide coordinated DNA methylation events and the implications in multiple cancers. Recently, analysis on co-methylation patterns has been performed on human brain and blood samples [14] using the same method as the weighted gene co-expression analysis [15]. This approach has led to discovery of an interesting module of co-methylated CpG islands and genes that are strongly associated with aging. Due to many factors that heavily affect DNA methylation levels and patterns, it is generally believed that cancer methylome is specific to specific tissue type [3]. However, the co-methylation study by Horvath [14] provides a new angle suggesting the possible existence of ubiquitous methylation patterns in multiple tissues. Therefore an important question is: are there such ubiquitous methylation patterns in cancers? If they exist, what are their roles? To be more specifically, do co-methylation clusters ever exist in different cancer tissue types in populations with mixed ages? If so, how they are related to cancer physiology, development and patient clinical outcomes?

In this study, we performed the first pan-cancer co-methylation cluster mining using our newly developed lmQCM algorithm [16], which has been successfully applied to gene co-expression analysis. For the first time, we identified four co-methylated clusters in multiple cancer types. Among the four clusters identified, two of them clearly separate the cancer from normal samples in 10 out of 11 cancer types. We also found that although the majority of tumor suppressors and oncogenes may be stably repressed/active in cancer cells as indicated by stable methylation levels, some genes that are commonly believed to be silenced are not universally silenced in mixed cancer-type populations.

## Results

### Compare co-methylation clusters in different cancer types

DNA methylation is known to be heavily affected by environmental factors [17], such as age, cell type and cell developmental stages [1, 18]. And cancer genome were often thought to be generally hypomethylated [19, 20]. However, despite the generally low methylation, highly diverse backgrounds of the TCGA cancer patients, as well as the highly diverse cell types, by applying our two-step frequent cluster mining workflow as described in the Methods section, we are able to identify recurring co-methylated clusters in different cancer types with different data platforms and different tumor to normal compositions using 17 datasets from 11 tumor types from TCGA (for details, see Table 1). These frequently identified co-methylation clusters may indicate common gene regulations in different cancer types. Among them, 17,181 pairs of co-methylated probesets are detected in over 50% of the cancer datasets we analyzed, which involve over 800 genomic regions. The array platform difference has minimal effect on the identified co-methylation clusters for AML, LUSC, STAD and UCEC data, but it showed some discrepancies for GBM and COAD data (Table 1). Since there is no data from the Illumina-27 platform for CESC, LIHC and THCA, lmQCM mining on that platform was not performed for these three datasets (listed as “NA” in Table 1).

**Table 1:** TCGA multiple cancer DNA methylation dataset and co-methylation mining summary. The last column displays “number of clusters in common cluster pairs from platform 1/number of clusters in common cluster pairs from platform 2/number of common cluster pairs”

Dataset	Total Sample#	Total Normal#	HM-27 sample (normal)	lmQCM clusters	Probe pair (unique pair) in all clusters	HM-450 Sample (normal)	lmQCM clusters	Probe pair (unique pair) in all clusters	Common clusters between platforms
GBM	450	2	295(0)	11	812429(807245)	155(2)	11	968165(967951)	5/4/5
COAD	555	75	203(37)	13	1685392(1683052)	352(38)	8	917282(916822)	4/4/4
CESC	312	3	0	NA	NA	312(3)	13	812354(811157)	NA
OVCA	623	30	613(30)	24	351921(350936)	10(0)	NA	NA	NA
LIHC	430	50	0	NA	NA	430(50)	9	1059318(1057998)	NA
LUSC	573	69	161(27)	11	2735398(2735122)	412(42)	16	1402241	8/10/11
BRCA	343	27	343(27)	9	692142(688536)	0	NA	NA	NA
STAD	470	27	73(25)	7	6184477(6174821)	397(2)	11	2113303(2112253)	4/6/7
THCA	571	56	0	NA	NA	571(56)	9	116820(116799)	NA
UCEC	591	35	118(1)	8	1482759(1476728)	473(34)	17	629431(629159)	7/11/12
AML	388	0	194(0)	13	841743(839045)	194(0)	14	433671(430841)	11/13/14

**Fig. 1:**
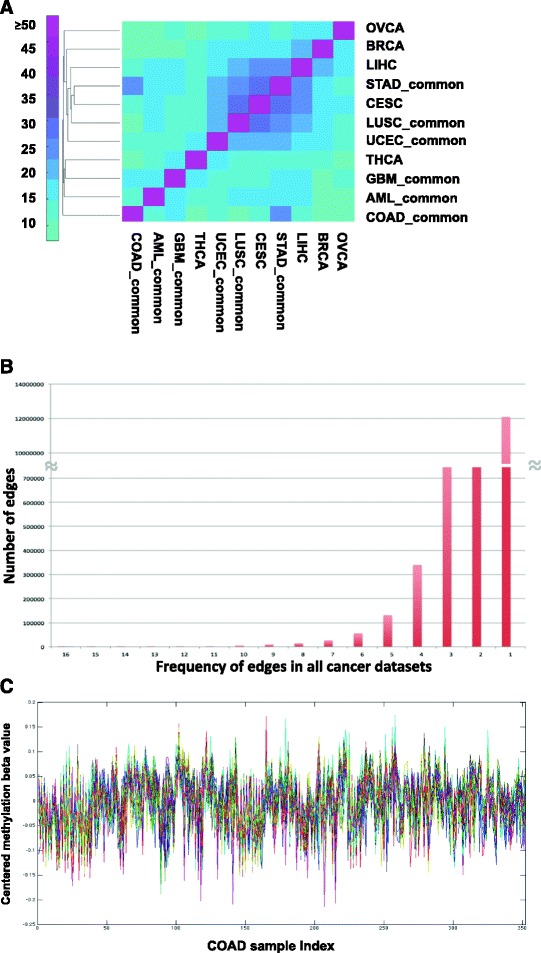
Similarity of co-methylation clusters among multiple cancer types, cluster edge frequency distribution among all cancer types, and an example of correlated methylation level within one cluster in COAD. **a** Clustered heatmap of Jaccard indices among co-methylation clusters in 11 cancer types, for the cancer types with two different methylation data platforms, only the common cluster probe pairs were used for comparison, as indicated by common after cancer type names. **b** The frequency distribution of the cluster edge (probe-pairs) in all 17 cancer datasets. **c** The centralized methylation beta values of Cluster 4 probes for all COAD-450 cohort. Colored lines represent different probes

Different cancer types also show distinctive co-methylated clusters of their own kind. To identify the cancer type specific co-methylation clusters and as well as common co-methylation clusters in multiple cancer types, we took a two-step approach. First, we were interested to find which cancer type is more unique in terms of co-methylated modules as compared to the other cancer types. By computing the Jaccard indices between every pair of cancer types coerced co-methylated clusters, we found that, in general, co-methylation clusters are mostly unique for OVCA and AML; while digestive system cancers STAD, COAD and LIHC co-methylation clusters are more similar to each other (Fig. 1a). However, Jaccard indices only compare co-methylation probe-pair lists between each pair of cancer types, thus will not reflect the commonality between cluster from each cancer type and the universal co-methylation clusters. Instead, we applied an additional step of frequent cluster mining using the same algorithm in the second step.

### Common co-methylated pan-cancer clusters

We pooled the co-methylated network edges (probe pairs) identified from all of the co-methylation clusters in 17 pan-cancer datasets for frequency counting (for the probe pair frequency distribution, please see Fig. 1b). By using the frequency as weight, we identified for the first time that universal co-methylation modules exist in different cancer types and we identified four such clusters in 11 cancer types studied (for a complete list of the four cluster genes, please see Additional file 1: Table S1).

**Fig. 2:**
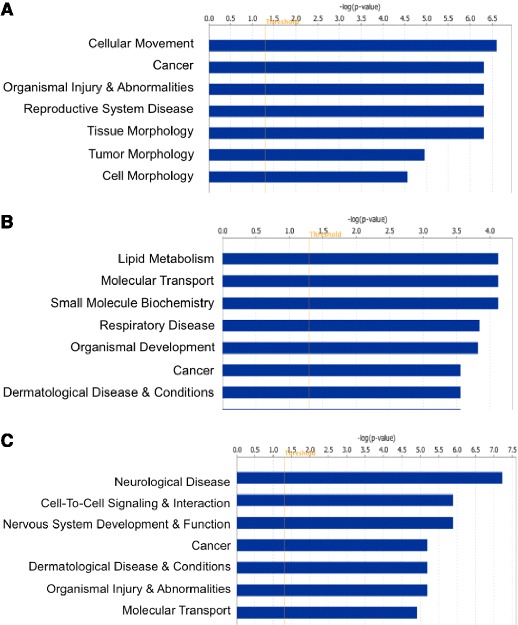
Top enriched biological functions for Cluster 1, 3 and 4 marked genes using Ingenuity Pathway Analysis (IPA). **a** Cluster 1 genes enriched biological functions. **b** Cluster 3 genes enriched biological functions. **c** Cluster 4 genes enriched biological functions

Cluster 1 contains genomic locations that involved 81 genes or gene families, and is the largest cluster in the four. IPA analysis showed that genes in this cluster are mostly involved in cellular movement, cell signaling, tissue morphology and cellular development (Fig. 2a). This cluster contains one methyltransferase BHMT and a group of kinases, kinase receptors and other membrane proteins, which are more likely to be involved in tumor microenvironment. However, only one gene CDH1 has been considered previously as cancer gene in Sanger Institute Cancer Gene Census.

Cluster 2 marked 31 gene/gene family regions that are all located on X chromosome. This is not surprising. Since many homologous genes on X chromosome are methylated for repression, especially for women where one of the X chromosomes is condensed and genes on it are repressed (X-chromosome inactivation), the QCM algorithm will naturally identify co-methylated clusters from those randomly hypermethylated regions on X chromosome. Five of our 17 datasets consist only female patients, which are UCEC (two datasets), OVCA, BRCA and CESC. These datasets will contribute X-chromosome methylation clusters to the pan-cancer study. Still inside the X-chromosome cluster, there are a few cancer-related genes (ATRX, MTCP1, PHF6), but the majority of the genes should be just the results of the X-chromosome inactivation. Mainly housekeeping biological functions are enriched in this cluster, and since all genes are from X-chromosome, the cluster is enriched with X-linked hereditary diseases. Although Cluster 2 may not provide too much information in term of cancer, it serves as a great internal positive control to demonstrate that our lmQCM workflow performed well on DNA co-methylation cluster mining.

**Fig. 3:**
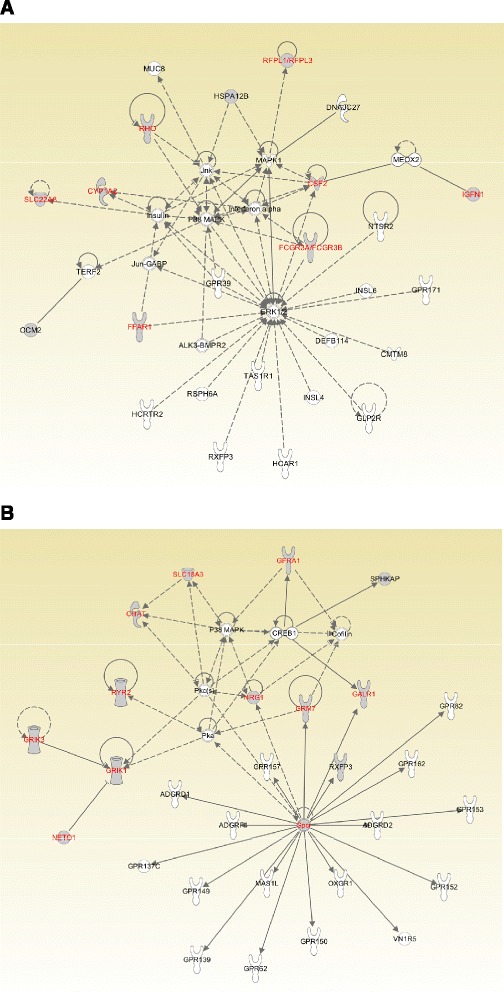
Top networks identified with IPA for cluster 3 and cluster 4 marked genes. **a** Cluster 3 cell signaling, molecular transport, vitamin and mineral metabolism network. Names in red: genes involved in cell signaling and cancer. **b** Cluster 4 cell signaling and interaction, nervous system development and function, neurological disease network. Names in red: genes involved in cell signaling and neural signal transmission. Grey: genes from Cluster 3 or 4. White: molecules not present in Cluster 3 or 4

Cluster 3 marked 26 genes/gene families, which includes cell signaling genes and immune response genes (Figs. 2b and 3a). Cluster 4 with 25 genes/gene families contains mostly nervous system genes and cell-to-cell signaling genes (Figs. 2c and 3b). However, only about one third of the genes from either cluster form known networks, as seen from Fig. 3ab. Majority of them were not functionally linked previously.

In every cancer type, the methylation levels of the genes in these four clusters are summarized into four eigengenes using the method described in the Methods section. Figure 1c shows the highly correlated beta values of DNA methylation from Cluster 4 among COAD-450 patients, with each color line representing one gene from Cluster 4.

Previous studies showed that DNA methylation in cancer is usually low on tumor suppressors and high on oncogenes [1, 18, 21, 22]. In our co-methylation study, we are looking for co-varied methylation regions, therefore not many tumor suppressor or oncogenes showed up in the pan-cancer co-methylation clusters, presumably due to lack of methylation variability of those genes among patients. Using available tumor suppressor database, we found only 2–6% of the tumor suppressors are found in any of the cancer-specific clusters in BRCA, COAD, LUSC, THCA and UCEC (Additional file 1: Table S2).

### Relationships between frequent co-methylation clusters and cancer types

**Fig. 4:**
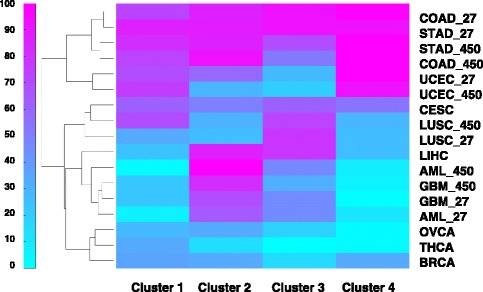
Contributions of edges to the four frequent co-methylation clusters from each of the 11 cancer types. The percentages of shared edges with respect to the total number of edges in each of the four clusters are plotted in the heatmap

**Fig. 5:**
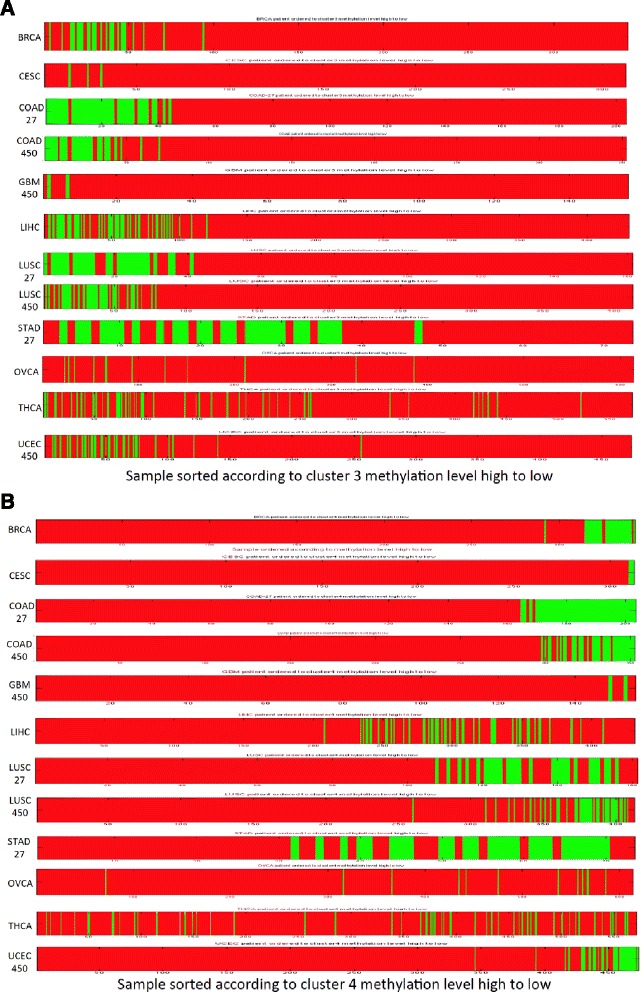
Samples differentiated by the transformed methylation level of Cluster 3 and 4 eigengene. Green: normal samples; Red: tumor samples. **a** sorted by Cluster 3 eigengenes for the methylation beta value from high to low. **b** sorted with Cluster 4 eigengenes for the methylation beta value from high to low

We also studied how different types of cancer differ in their contributions in the four frequent co-methylation clusters. The results are shown as a clustered heatmap in Fig. 4. We found that THCA has the most unique co-methylation pattern than all other cancer types and contributes the least to the four frequent co-methylation clusters, followed by OVCA and AML. The relatively younger age of THCA patients may partially account for the methylation difference (Fig. 5). It was reported that AML methylation was different from the solid tumor, and this study confirmed that finding [23]. In contrast, most of the pan-cancer clusters can be found in COAD co-methylated clusters, so is in STAD. Especially for Clusters 3 and 4, over 90% of probes are found in COAD and STAD clusters mined from HM 27 K array data.

### Co-methylated pan-cancer clusters show difference between normal and cancer samples

The most interesting findings are that in 10 out of the 12 cancer datasets with normal samples, the samples were clearly separated by tumor and normal type according to the methylation levels of the eigengenes for probe regions of the Clusters 3 and 4 (Fig. 5a and b). In Fig. 5a and b, we sorted the methylation levels from high to low for the eigengenes of these two clusters. As shown in Fig. 5a, Cluster 3 marked genes are heavily methylated in the normal samples, but less methylated in tumor samples, whereas in Cluster 4 it is the opposite --- the tumor samples are heavily methylated, and the normal samples are less methylated. This separation appears to be universal in epithelial cancer types, since nine out of ten cancer type showed this trend. Unfortunately, there is no normal samples in AML and very few in GBM, so it will be our future work to test if this holds true for non-epithelium derived cancer.

This separation also depends on how many probes are shared between Cluster 3 (or Cluster 4) with the cancer-specific clusters in each cancer type. For example, for STAD and COAD, which over 90% of their co-methylation clusters are shared with the pan-cancer clusters, have the most clear-cut separation, while THCA samples, which has the minimal overlap (0 and 2.85%) with Clusters 3 and 4 showed no separation of tumor and normal along the eigengenes’ values.

Since tumor suppressors are believed to be highly methylated while oncogenes to be least methylated in cancer cells [18, 21, 22], we checked if any of these two categories of genes in Clusters 3 or 4 following that pattern. There was only one tumor suppressor (CSF2) and one cancer census gene in Cluster 3 (USP6); there are four tumor suppressors (GALR1, IRF4, PTPRT and SOX11) and one more cancer census gene in Cluster 4 (NRG1). This may indicate that the majority of the cancer-related genes (suppressors or oncogenes) are stably methylated/unmethylated among the tumor samples or only differentially methylated in some specific type of cancers, therefore not present in the frequent co-methylation clusters. However, the majority of genes we identified in Clusters 3 and 4 are not linked with cancer. They may be the potential biomarkers to differentiate cancer from normal tissue as well stratify patient disease subtypes/stages, or provide directions for future cancer research.

### Relationship between co-methylation clusters and aging

**Fig. 6:**
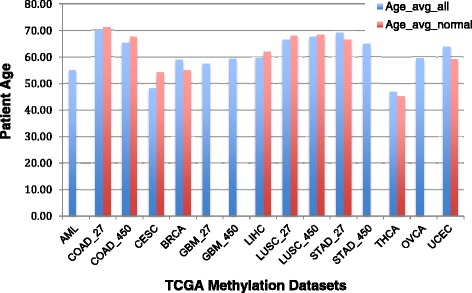
Age average in multiple cancer datasets used for co-methylation cluster mining. Blue: age average for all samples. Red: age average for normal samples

Since age is a major factor affecting DNA methylation patterns, we also examined the age distribution in all analyzed cancer datasets and found that THCA and AML datasets consist of the youngest population of patients, which may partially explain the unique co-methylation patterns in these two datasets when compared to all other cancer types (Fig. 6). However, CESC data also contain relatively young population of patients, the co-methylation pattern is not as distinctive as those in THCA and AML. We speculate that other factors such as cell type may also play a role in the co-methylation profile in AML and THCA. TCGA normal samples are usually adjacent normal tissues from the same cancer patient. Therefore age is not likely to contribute to the separation of tumor/normal samples as we observed in the Clusters 3 and 4 sorted methylation profiles (Fig. 5ab). To investigate if age is involved in the sample differentiation as observed in Fig. 5, we plotted the average ages of entire cohort as well as normal cohort for each type of cancer. Not surprisingly, we did not find significant age difference between entire cohort and normal sample cohort, therefore our findings of tumor vs. normal separation in cluster 3 and 4 genes were not due to age factor (Fig. 6).

**Fig. 7:**
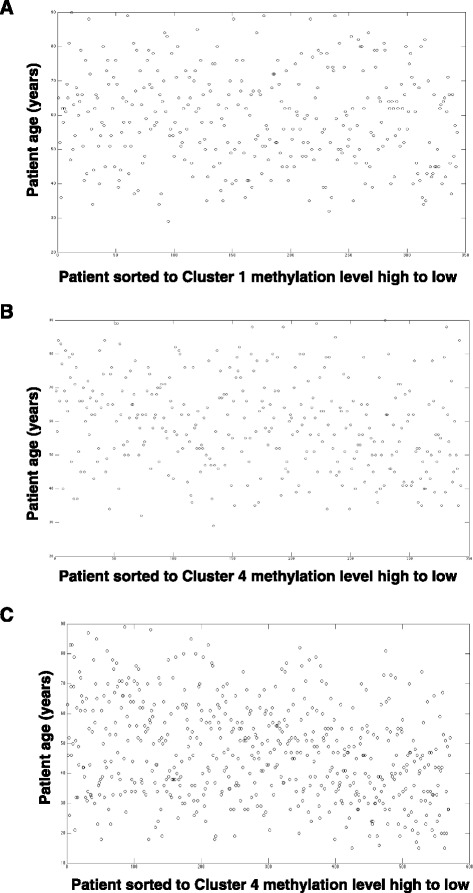
Patient ages sorted according to the transformed methylation values of the eigengenes for Cluster 1 and Cluster 4 of BRCA and THCA. **a** patient age sorted according to methylation levels of the eigengene for Cluster 1 from high to low in BRCA. **b** patient age sorted according to Cluster 4 eigengenes' methylation level high to low in BRCA. **c** patient age sorted according to Cluster 4 eigengenes' methylation level high to low in THCA

In addition, we examined if any of the four pan-cancer clusters’ methylation level correlates with age. We checked all of the cancer types analyzed in this project. The results showed no correlation of age with the level of methylation of the four clusters. Three of them in BRCA and THCA are shown in Fig. 7.

## Discussion

It is widely accepted that tumor suppressors are hypermethylated in their promoter region and repressed in tumor samples [8, 21, 22]. The hypermethylation usually leads to silencing of the genes and a list of commonly repressed and silenced genes from five types of cancer (BRCA, LUCA, PC, Leukemia and CRC) can be found in literature [1]. However, in this study, we found that majority of them showed highly variable methylation level in cancer cells, even without normal control (Table 2). This is not surprising, since many cancers were found to have distinctive CIMP subgroups [3]. Our findings confirmed this and expand the knowledge from the current view of a few genes’ methylation patterns change to more coordinated changes of methylation changes in a network fashion which is universal to multiple cancer types. On one hand, this result implies clinicians should be cautious when using the methylation genes as biomarkers for diagnosis, while on the other hand, it provides new directions to apply these genes for tumor subtyping.

**Table 2:** Crosscheck of cancer-specific co-methylation clusters with genes silenced by promoter hypermethylation in multiple cancer [1, 2, 25, 33, 34]

	AML	BRCA	COAD	CESC	GBM	LIHC	LUSC	STAD	THCA	OVCA	UCEC
APC					X						
BMAL1											
BRCA1									X		X
CDH1	X	X		X		X	X	X	X	X	X
CDH13	X							X			X
CDKN2A		X									
CDKN2B		X									
COX2											
CRABP1			X								
DAPK1								X			X
ESR1											X
GATA5			X				X				
GSTP1							X	X	X		
HIC1											
IGFBP3			X								
MGMT	X								X	X	X
MLH1											
NORE1A											
P14											
PYCARD		X									
RARB2											
RASSF1A											
TLE1											
TP73	X			X			X				

X indicates a hit in corresponding cancer-specific co-methylation clusters

One of the major finings in this study is the existence of pan-cancer co-methylation clusters. Co-methylated gene clusters from normal cell lines [24] have been studied using the WGCNA package [15] recently, which is complementary to our pan-cancer study. Pan cancer study of DNA methylation patterns [12] were also carried out using differential methylation analysis. However, our work described the first co-methylation cluster mining with correlated variable methylation states on a pan-cancer scale. The finding of two tumor suppressor genes BVES and PRDM1 shared by all 15 cancer types in [12] was not found in any co-methylation clusters of the 11 cancer types, presumably due to these sites being constant hypermethylated and lack of variation.

There are four major co-methylation clusters among the 11 types of cancer studied. In the largest frequent co-methylation Cluster 1, many genes (such as BHMT, CSNK1E, CTSZ, CXCL1, CXCL17, CDH1, ERBB2, GAK, GGT1, GPR56, HBEGF, HNF1A, ICAM3, IL17RC, IL22RA1, NCOR2, OSM, PHKG1, PTPRCAP, TNFAIP8L2) encode enzymes, ligands, receptors or transcription factors/repressors that are involved in DNA methylation, cell-to-cell signaling and development. However, the 81 genes in this cluster are not tightly connected based on current knowledge (Query in STRING database, Additional file 1: Figure S1). The methylation levels of these genes were not correlated with available patient clinical traits or age either (Fig. 7a). Thus the underlying correlated methylation pattern and the underlying molecular mechanism is yet to be elucidated. Among them, only CDH1 was previously considered to be cancer epigenetic-biomarkers, and reported silenced by hypermethylation in breast, colon, lung, leukemia and prostate cancer (Table 2, [1]). However, a highly variable methylation level of CDH1 was observed in this study in multiple cancers by its presence in frequent co-methylation cluster, as well as in cancer-specific co-methylation clusters from AML, BRCA, CESC, STAD, LUSC, LIHC, THCA, OVCA and UCEC, especially it is present in AML, CESC and STAD where there is zero or 2 to 3 normal tissue samples.

The most exciting discovery of the frequent co-methylation clusters is the separation of tumor versus normal samples by Clusters 3 and 4, which shows opposite methylation trends between tumor and normal samples. Again this pattern is not correlated with age at all in any cancer type (Fig. 7bc). This finding suggests that despite distinctive tissue specific epigenomic patterns, there are consistent and ubiquitous changes between tumor and normal tissues. Interestingly, currently there is no known methylation biomarkers for cancer in Cluster 3 or 4, nor were majority of those genes linked with cancer previously. Our results thus provide new insights and potential candidates for cancer patient stratification biomarkers and therapeutic targets.

In colon cancer, DNA methylation was linked to phenotype stratification more than fifteen years ago [7] and quite a few genes have been identified as DNA methylation biomarkers since then [25]. Among them, CRABP1, FLNC, IGFBP3, MIR34B, MYOD1, RUNX3, SFRP1 and SFRP2 are found to be co-methylated in the same cluster in both of the TCGA colon adenocarcinoma datasets. However, none except CDH1 of the above biomarkers was found in the frequent co-methylation clusters, suggesting these DNA methylation biomarkers are more specific for colon cancer type.

The loss of imprinting of IGF2 and H19 from parental alleles were previously linked to cervical cancer; Loss of imprinting of H19 was also linked to lung cancer and hepatoblastoma [26]. In our study, we found IGF2 and its receptor IGF2BP2/3 in co-methylation clusters from 12 out of 17 datasets (BRCA, LUSC, CESC, STAD, THCA, UCEC, AML, OVCA), and H19 in the co-methylation cluster from STAD and BRCA. The variable level of methylation levels of these two genes along with CDH1 gene may indicate the heterogeneity of cancer population in most cancer types in TCGA database. Indeed, it has been reported that different DNA methylation patterns can be used to subtyping TCGA breast cancer patients [26, 27], and in AML [28, 29]. In AML, when we compared the published gene list of 45 genes that are aberrantly methylated in all AML subtypes, there is no overlap between those genes and the frequent co-methylation clusters, and only four genes out of 1109 genes were shared between that list and our AML-specific co-methylation clusters (BTBD3, CYP26C1, MCTS1, ZFP161), indicating that most of the AML-linked genes are either not highly variable in their methylation levels or not part of the co-variation network in the TCGA cohort we examined.

## Conclusion

To summarize, with the accumulation of large genomic datasets such as TCGA, we are able to carry out pan-cancer and comparative studies on epigenetic markers, which have not been previously examined. By applying the newly improved lmQCM algorithm and the similar workflow as in gene frequent co-expression network mining, we successfully identified four frequent co-methylation clusters from 11 cancer types, and two of them can clearly separate the tumor samples from normal samples based on their methylation levels. It demonstrated from the first time that consistent epigenetic landscape changes exist in multiple cancer types. The results from this study lead to interesting biological question on the molecular mechanism for co-methylation, while at the same time will provide insights and new directions for potential cancer epigenetic marker and therapeutic target findings.

Currently, we are extending the same workflow to other cancer DNA methylation data to verify the findings of these frequent co-methylation clusters and examining the predictive power of differentiating cancer and normal samples by clusters 3 and 4.

## Methods

### Data acquisition and processing

Seventeen (17) DNA methylation datasets and the corresponding clinical data for 11 different cancer types were downloaded from The Cancer Genome Atlas (TCGA) data portal (https://tcga-data.nci.nih.gov/tcga/) in January 2016. The details of sample information for each cancer dataset are summarized in Table 1. Level-3 DNA methylation beta values were consolidated and filtered for each cancer dataset. To remove noise and probesets with low information content, probesets without values or with variance lower than 30 percentile of the variance across the entire probesets in each dataset were removed, then probesets with average values across the entire cohort lower than 20 percentile of all the mean values were removed. Among the 11 cancer types, colon adenocarcinoma (COAD), acute myeloid leukemia (AML), lung squamous cell carcinoma (LUSC), stomach adenocarcinoma (STAD), uterine corpus endometrial carcinoma (UCEC) and glioblastoma multiforme (GBM) each contain two sets of DNA methylation data, namely the Illumina Infinium HumanMethylation 27 K Beadarray and 450 K Beadarray datasets. They were processed separately, and annotated with “−27” or “−450” after the cancer types. In Illumina 450 K array data, only probes with matching 27 K array probes were used, so clusters obtained can be compared with each other. Datasets with fewer than 50 samples were excluded from the analysis. Also if both platforms are present for any specific cancer, since the patients and samples are different in different platforms, we consider each dataset separately and include both to construct a larger data pool for frequent network mining,

### Frequent co-methylation cluster mining in multiple cancer datasets

The workflow to mine frequent co-methylated clusters was adapted from our previously established workflow for frequent gene co-expression clusters in multiple cancers using quasi-clique merging (QCM) algorithm [30]. The steps are summarized as following:

1. Mine co-methylated clusters on processed DNA methylation data (including both tumor and normal data if available) using newly improved lmQCM algorithm [16] on weighted co-methylation network for each cancer dataset. lmQCM algorithm decreases the bias towards the high densely connected clusters in QCM by first normalizing the weight matrix. Specifically, the co-methylation network was established with the absolute values of the Spearman correlation coefficient (SCC) being the weight of the edges. Then the weights were transformed such that the sum of the columns and the rows are all one using the same iterative procedure for normalizing the graph Laplacian matrix (with the sums of rows and columns being 1) as in spectral clustering [31]. This procedure can reduce the bias caused by highly connected clusters and enable the algorithm to detect more subtle clusters. Then the clusters were mined using a greedy approach as described in [30]. There are four parameters in the algorithm with ***γ*** being the major parameter controlling the threshold for the weight of the initial edge for new clusters. If among all the edges that have not been any part of the identified clusters, none of them has weight more than ***γ***, the algorithm will stop. Two other parameters ***λ*** (***λ*** ≥ 1) and ***t*** (***t*** ≥ 1) controls the decreasing rate of the densities of the detected clusters. While the densities of the clusters keep decreasing, the algorithm guarantees a lower bound of the cluster density [30]. The parameter ***β*** decides the maximum allowed overlap ratio between any two clusters. If two clusters have overlap ratio higher than ***β***, they will be merged into one cluster. Here the overlap ratio is defined as the ratio between the size of the intersections between two clusters and the size of the smaller cluster. Previously we found that for most of the networks we tested (including both gene co-expression networks and co-methylation networks), the number of output clusters is relatively stable between ***γ*** = 0.4 to 0.6, while choosing ***γ*** = 0.6 controls the size of the clusters. Similarly ***β*** = 0.4 balances between number of clusters and cluster sizes. The other two parameters ***λ*** and ***t*** do not have significant impact on the cluster number so we chose ***λ*** = 1.0, ***t*** = 1.0. In addition, we focus on relatively large clusters and thus set the minimum cluster merging size to be 20.
2. Generate probe pairs for each cluster obtained from each cancer dataset, assuming within each cluster every two probes were connected, then merged all of the probe pairs from all 17 datasets together and compute the frequency of every probe pair in the combined data. Use frequencies as weights we established a weighted network, which was mined to identify frequently co-methylated clusters. The clusters identified are called pan-cancer co-methylation clusters in this study. The parameter settings are the same as in step 1, except ***γ*** = 0.5 to ensure that every frequent cluster was initiated with at least one edge that appears in at least half of the datasets.

### Calculating “eigengene” using singular value decomposition for every cluster for each cancer dataset

The probes from each of the four identified clusters in step 2 in the workflow above were mapped back to each cancer dataset to obtain corresponding DNA methylation values. The values for each cluster were then summarized into an “eigengene” using the singular value decomposition approach as described by Langfelder and Horvath [32] based on singular decomposition. We implemented this procedure in MATLAB. The resulted eigengene represents a weighted average of the methylation levels for the probesets over the patients. It concentrates along the highest variable direction of the data vectors. Patient information was then extracted and compared between the tumor and normal samples. The values of each eigengene were also sorted in the order from high to low and displayed with tumor and normal annotations labeled in different colors (Fig. 5). Twelve datasets from 10 cancer types were analyzed. AML and GBM-27, which have no normal control samples, and STAD-450, which has only 2 normal samples, were not included in this sorting and comparison.

### Gene ontology enrichment analysis

Ingenuity Pathway Analysis (IPA) was used for probe-matched genes from the pan-cancer co-methylation clusters. For each of the four identified clusters, the probe corresponding genes were combined to generate a gene list. If a family of genes were matched to a single probe ID, only the first member of that family was included in the gene list to avoid enrichment bias. A core analysis was performed on each gene list in IPA. STRING database (http://string-db.org) was used to query protein-protein interactions for genes in frequent co-methylation clusters using the default settings.

### Comparison of co-methylation clusters between different platform and different cancer types

Co-methylation clusters identified from each cancer dataset were mapped to genes. The genes marked by such clusters of each cancer dataset were coerced to a single gene list to compare across different cancer types. Jaccard indices were generated for each pair of cancer type gene lists. For clusters generated from different data platforms (Illumina HumanMethylation 27 K or 450 K array) from the same cancer type, the genes marked by each cluster are compared, and the clusters with overlap genes 25% or more were considered to be common clusters (Table 1), and the common genes were pooled together and used to compute Jaccard indices between different cancer types. Here “-common” is used after the cancer type to denote the common clusters between two platforms in a specific cancer type (Fig. 1a).

The edges from the four pan-cancer co-methylation clusters were mapped back to original clusters identified from each cancer type to obtain the overlapping percentage with respect to the cluster edge number in a particular cancer clusters (Fig. 4). To visualize the clustering of cancer types using heatmaps, hierarchical clustering tool *clustergram* from MATLAB is used on original values of percentage (Fig. 1a) or Jaccard indices (Fig. 4) with default *average* linkage.

### Crosscheck with tumor suppressor gene, oncogenes, cancer gene census and published cancer methylation biomarkers

One thousand two hundred seventeen protein coding and non-coding tumor suppressor genes were obtained from Tumor Suppressor Gene Database, (https://bioinfo.uth.edu/TSGene/). 208 oncogenes were obtained from Ingenuity Pathway KnowledgeBase, and 571 genes of Cancer Gene Census were downloaded from COSMIC (Catalogue of Somatic Mutations in Cancer) website (http://cancer.sanger.ac.uk). Cancer type specific DNA methylation biomarkers were obtained from corresponding publications and crosschecked with both frequent co-methylation clusters and cancer-specific co-methylation clusters. If two types of array data for the same cancer type were studied, the common clusters between the two types were used for crosscheck with these lists [1, 2, 25, 33, 34].

## Additional file

Additional file 1: Table S1 Frequent co-methylation clusters. **Table S2.** Cross-check of known tumor suppressor with corresponding cancer co-methylation clusters. The numbers indicate the overlaps between co-methylated clusters and known tumor suppressor in each corresponding cancer type. Freq ≥ 9 genes were obtained from combined co-methylated clusters from all 17 cancer datasets and extracted the genes appeared in over 9 datasets. **Figure S1.** Protein-protein network query on STRING database for Cluster 1 genes. (DOCX 375 kb)

## Abbreviations

AML: Acute myeloid leukemia
BRCA: Breast invasive carcinoma
CESC: Cervical squamous cell carcinoma and endocervical adenocarcinoma
COAD: Colon adenocarcinoma
GBM: Glioblastoma multiforme
LIHC: Liver hepatocellular carcinoma
LUSC: Lung squamous cell carcinoma
OVCA: Ovarian serous cystadenocarcinoma
STAD: Stomach adenocarcinoma
THCA: Thyroid carcinoma
UCEC: Uterine corpus endometrial carcinoma
