# Supplementary material for: Pan-cancer analysis of frequent DNA co-methylation patterns reveals consistent epigenetic landscape changes in multiple cancers
Source: BMC Genomics. 2017 Jan 25;18(Suppl 1):1045. doi: 10.1186/s12864-016-3259-0 (PMC5310283; doi:10.1186/s12864-016-3259-0)

**Additional file 1 for “Pan-cancer analysis of frequent DNA co-methylation patterns reveals consistent epigenetic landscape changes in multiple tumors”**

Jie Zhang, Kun Huang

The Ohio State University, Department of Biomedical Informatics

**Table-S1 Frequent co-methylation clusters**

| Cluster 1 | Cluster 2 | Cluster 3 | Cluster 4 |
| --- | --- | --- | --- |
| BHMT  BIN2  BTBD8  C10orf27  C11orf52  C16orf54  C1orf210  C1orf66  C20orf151  C9orf139  CD3EAP  CD6  CDH1  CHRM1  CSNK1E  ISG20L2  CTSZ  CX3CL1  CXCL17  EDN2  ERBB2  ESRP1  ESRP2  EXOSC7  FAM113B  FAM78A  FUT7  FXYD3  GAK  GGT1  GPR56  GRB7  HBEGF  HNF1A  ICAM3  IFFO1  IL17RC  IL22RA1  INPP5J  BHMT  PCDHGA4  ITGB6  KLHL6  KLK6  KRT8  LOC100233209  MED22  MTMR11  NCOR2  OCLN  OSM  PAQR6  PART1  PARVA  RDH5  PDE4D  PHKG1  PLA2G4E  PPP1R13L  PRELP  PSORS1C1  PTGFRN  PTPRCAP  PVRL4  SERPINB5  RPL7A  SLC44A2  SCGB1D1  SFTA2  SHROOM1  SLC25A15  SNORD24  ZDHHC3  SOX9  STAP2  SULT2B1  TARBP1  TMEM149  TMEM175  TNFAIP8L2  TNKS1BP1 | ABCB7  ABCD1  AIFM1  ARMCX3  ATP6AP1  ATRX  BCAP31  BEX2  BRCC3  CD99L2  CXorf42  FAM3A  GPRASP2  HTATSF1  IDH3G  MAGT1  MTCP1  MTMR1  NKAP  OCRL  PGK1  PHF6  PHKA1  PLS3  RBMX  RPL36A  SLC10A3  SRPX  SSR4  UTP14A  VBP1 | C3orf22  CEACAM4  CSF2  CYP1A2  FCGR3B  FFAR1  HIPK4  HSPA12B  IGFN1  KRT76  KRT82  LACRT  LCE3D  OCM2  OR12D2  OR12D3  PPY  RFPL1  RFPL1S  RHO  SCN4A  SLC22A8  SPRR2A  TBC1D3C  USP6  WNT8A | BNC1  CHAT  CYP26B1  FERD3L  GALR1  GFRA1  GJD2  GRIK1  GRIK3  GRM7  HS3ST2  IRF4  KCNJ6  LMX1A  LPPR3  NETO1  NRG1  PTPRT  RXFP3  RYR2  SLC18A3  SOX11  SPHKAP  VWC2  WNT2 |

**Table-S2 Cross-check of known tumor suppressor with corresponding cancer co-methylation clusters.** The numbers indicate the overlaps between co-methylated clusters and known tumor suppressor in each corresponding cancer type. Freq≥9 genes were obtained from combined co-methylated clusters from all 17 cancer datasets and extracted the genes appeared in over 9 datasets.

| **Co-methylation clusters in specific**  **Cancer dataset** | | **COAD**  **clusters** | **BRCA clusters** | **LUSC**  **clusters** | **THCA** | **UCEC**  **clusters** | **Freq ≥9 genes** |
| --- | --- | --- | --- | --- | --- | --- | --- |
| **Published**  **Tumor**  **suppressors** | **Total genes** | 688 | 1828 | 1332 | 881 | 991 | 801 |
| **COAD** | 535 | 40 |  |  |  |  |  |
| **BRCA** | 589 |  | 78 |  |  |  |  |
| **LUSC** | 534 |  |  | 41 |  |  |  |
| **THCA** | 486 |  |  |  | 25 |  |  |
| **UCEC** | 526 |  |  |  |  | 29 |  |
| **All pan cancer study** | 983 |  |  |  |  |  | 46 |

**Figure-S1 Protein-protein network query on STRING database for Cluster 1 genes.**


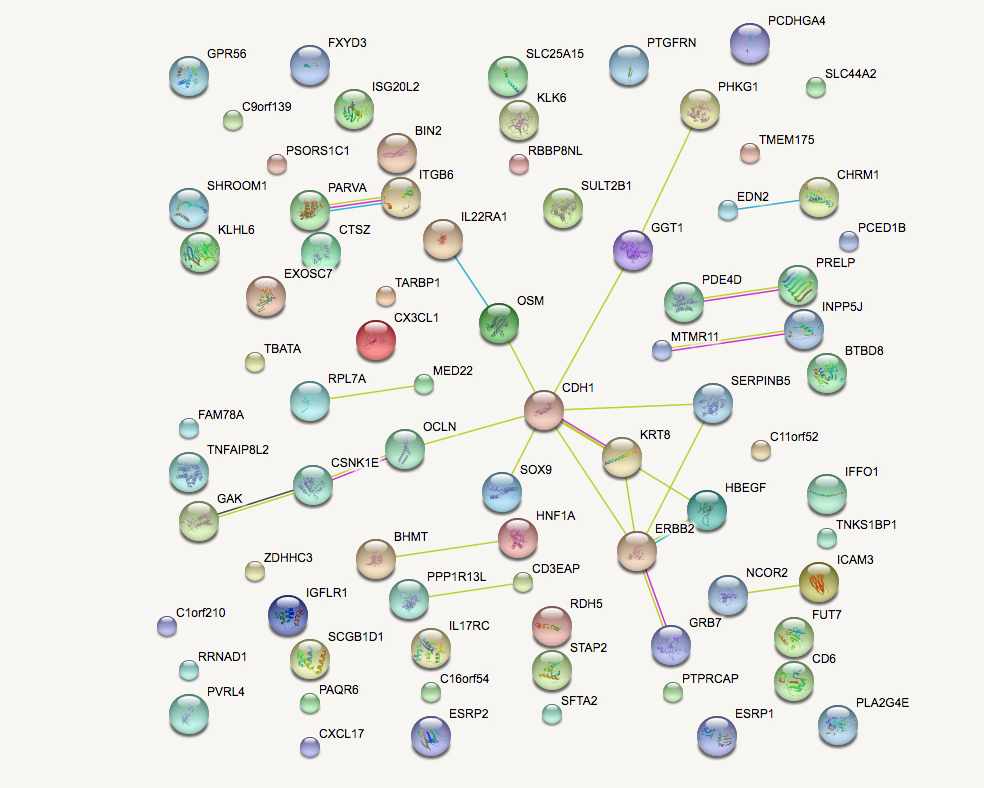


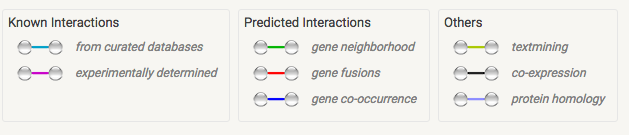

Supplement: Additional file 1: Table S1 — Frequent co-methylation clusters. Table S2. Cross-check of known tumor suppressor with corresponding cancer co-methylation clusters. The numbers indicate the overlaps between co-methylated clusters and known tumor suppressor in each corresponding cancer type. Freq ≥ 9 genes were obtained from combined co-methylated clusters from all 17 cancer datasets and extracted the genes appeared in over 9 datasets. Figure S1. Protein-protein network query on STRING database for Cluster 1 genes. (DOCX 375 kb) [file 12864_2016_3259_MOESM1_ESM.docx]
